# Human Sensory Neurons Derived from Induced Pluripotent Stem Cells Support Varicella-Zoster Virus Infection

**DOI:** 10.1371/journal.pone.0053010

**Published:** 2012-12-28

**Authors:** Katherine S. Lee, Wenbo Zhou, Jonah J. Scott-McKean, Kaitlin L. Emmerling, Guang-yun Cai, David L. Krah, Alberto C. Costa, Curt R. Freed, Myron J. Levin

**Affiliations:** 1 Department of Pediatrics, Section of Infectious Diseases, University of Colorado Denver, Aurora, Colorado, United States of America; 2 Neuroscience Training Program, Division of Clinical Pharmacology and Toxicology, Department of Medicine, University of Colorado Denver, Aurora, Colorado, United States of America; 3 Merck Sharp & Dohme Corporation, West Point, Pennsylvania, United States of America; Cincinnati Children's Hospital Medical Center, United States of America

## Abstract

After primary infection, varicella-zoster virus (VZV) establishes latency in neurons of the dorsal root and trigeminal ganglia. Many questions concerning the mechanism of VZV pathogenesis remain unanswered, due in part to the strict host tropism and inconsistent availability of human tissue obtained from autopsies and abortions. The recent development of induced pluripotent stem (iPS) cells provides great potential for the study of many diseases. We previously generated human iPS cells from skin fibroblasts by introducing four reprogramming genes with non-integrating adenovirus. In this study, we developed a novel protocol to generate sensory neurons from iPS cells. Human iPS cells were exposed to small molecule inhibitors for 10 days, which efficiently converted pluripotent cells into neural progenitor cells (NPCs). The NPCs were then exposed for two weeks to growth factors required for their conversion to sensory neurons. The iPS cell-derived sensory neurons were characterized by immunocytochemistry, flow cytometry, RT-qPCR, and electrophysiology. After differentiation, approximately 80% of the total cell population expressed the neuron-specific protein, βIII-tubulin. Importantly, 15% of the total cell population co-expressed the markers Brn3a and peripherin, indicating that these cells are sensory neurons. These sensory neurons could be infected by both VZV and herpes simplex virus (HSV), a related alphaherpesvirus. Since limited neuronal populations are capable of supporting the entire VZV and HSV life cycles, our iPS-derived sensory neuron model may prove useful for studying alphaherpesvirus latency and reactivation.

## Introduction

Varicella-zoster virus (VZV) is a human alphaherpesvirus that has infected over 90% of people worldwide, causing varicella (chickenpox) as well as herpes zoster (shingles). After primary infection, VZV establishes lifelong latency in sensory ganglia. Reactivation of latent VZV leads to herpes zoster, which can arise spontaneously but occurs most often when the VZV-specific T cell-mediated immunity of an individual declines, either because of immune suppression or aging [1]–[4]. In the United States, the societal costs for treating patients with herpes zoster and its complications are estimated to exceed $1 billion annually [5].

In an effort to understand the mechanisms that control VZV reactivation and the pathogenesis of herpes zoster, there have been many attempts to develop in vitro and in vivo models. Difficulties in developing small animal models result from the strict species specificity of VZV, prompting studies of simian varicella virus infection in non-human primates. Although these infections possess clinical and virological characteristics that resemble those seen after VZV infection, such as the establishment of latency [6], [7], primate models are costly and not readily available. While engraftment of human tissues into the SCID-hu mouse model is useful for examining replication in different cell types [8], [9], the limited availability of tissues (e.g. dorsal root ganglia) for xenografts and technical challenges limits its application. Additionally, although the role of different viral genes important for VZV latency has been studied in a non-productive cotton rat model, it is not a natural host and therefore, the genes may not have the same functions in humans [10]. Candidate in vitro models include using neurons from aborted fetuses [11], [12] and from guinea pig enteric ganglia [13]–[15]. Multiple efforts have also been made to isolate primary neurons from adult human sensory ganglia, including dorsal root and trigeminal ganglia, to establish an in vitro model of infection and latency. However, obtaining preparations from humans present many logistical difficulties, and there are inherent genetic variations in neurons between human samples, hindering reproducibility of experimental results. Pathogenesis studies with guinea pig enteric neurons are based on a non-permissive host, which is likely to provide results substantially different from pathogenesis in humans. Recently, a differentiated neuroblastoma line was shown to support VZV infection [16], but cancer cell lines differ substantially from primary cells. Within the last year, two neuronal models of VZV infection have been described that are based on human neural stem cells [17] and human embryonic stem (ES) cells [18]. While these models have increased our understanding of the interactions of VZV with neurons, the cells that supported VZV infection were not specifically programmed and characterized as sensory neurons, which are known to be one of the few reservoirs of latent VZV.

The creation of induced pluripotent stem (iPS) cells [19], [20] may advance our understanding of multiple diseases and serve as a platform for therapeutic interventions [21]–[23]. iPS cells are derived from somatic cells that are reprogrammed by the introduction of key stem cell genes to become pluripotent cells that closely resemble ES cells. We previously generated human iPS cells from fibroblasts by the introduction of four reprogramming genes using non-integrating adenovirus [24]. There have only been a few studies directed at generating sensory neurons from either ES or iPS cells, with the majority of them focusing on peripheral nervous system diseases, such as familial dysautonomia [25]–[28]. Most of these studies used murine stromal cell lines, such as PA6 or MS5, that promote neural differentiation of stem cells via their “stromal cell-derived inducing activity” [29], [30], followed by the formation of either neural rosettes or neurospheres, a spherical cluster of neural stem cells [31], [32]. Neurospheres are renewable and multipotent, bypassing the need to begin each differentiation experiment with ES or iPS cells, but they are heterogeneous and their physical structure results in limited and unequal access to nutrients.

In the present report, we describe the conditions required for the reproducible differentiation of human iPS cells to sensory neurons. We show that these neurons express sensory neuron markers and are electrophysiologically active. Importantly, we demonstrate their capacity to support VZV infection, as well as infection by the related alphaherpesvirus, herpes simplex virus (HSV). Because sensory neurons are one of the only cell types that support the entire VZV life cycle, our model may be able to address questions regarding VZV latency and reactivation.

## Materials and Methods

### Tissue culture

Human embryonic fibroblast IMR90 cells (ATCC, Manassas, VA) were reprogrammed to induced pluripotent stem (iPS) cells using adenoviral vectors expressing c-Myc, Klf4, Oct4, and Sox2, as previously described [24]. Mitotically inactivated mouse embryonic fibroblasts (MEFs; Millipore, Temecula, CA) were seeded on gelatin-coated tissue culture plates and grown in Dulbecco's modified Eagle's medium (DMEM), 10% fetal bovine serum, and 1% penicillin-streptomycin-L glutamine (P/S/G). The next day, iPS cells were cultured on MEFs in the presence of DMEM/F12 (Life Technologies, Grand Island, NY), 20% knockout serum replacement (KSR, Life Technologies), 1% P/S/G, 1% nonessential amino acids, 0.1 mM β-mercaptoethanol, and 10 ng/mL basic fibroblast growth factor (bFGF). MRC5 fibroblasts were cultured in DMEM containing 10% FBS and 1% P/S/G. All cells were grown at 37°C and 5% CO_2_.

### Generation of sensory neurons

When iPS colonies reached 50–70% confluence, they were dissociated using Accutase (Innovative Cell Technologies, San Diego, CA) and incubated on a non-coated 10-cm tissue culture dish for 30 min at 37°C to remove any remaining MEFs. The supernatant containing iPS cells was carefully collected and centrifuged at 1000 rpm for 5 min. The cells were resuspended at a density of 2×10^4^ cells/well in DMEM/F12, 10% KSR, 1% P/S/G, 0.3 uM SU5402 (Tocris, Ellisville, MO), 1.0 uM RO4929097 (Cellagen Technology, San Diego, CA), 5 uM CHIR99021 (BioVision, Mountain View, CA), 1 uM A83-01 (Cellagen), 0.2 uM LDN-193189 (Cellagen) and 0.1 uM retinoic acid (Sigma-Aldrich, St. Louis, MO) and plated on Matrigel-coated 12-well plates. The media was replaced every 2–3 days. On day 10, the media was changed to DMEM/F12, 10% KSR, 1% P/S/G, 1× N2, neurotrophin-3 (NT-3; 10 ng/mL), brain-derived neurotrophic factor (BDNF; 10 ng/mL), nerve growth factor (NGF; 10 ng/mL), and glial cell line-derived growth factor (GDNF; 10 ng/mL), ascorbic acid (200 uM) and 0.5 mM dibutyryl cAMP. The media was replaced every 2–3 days for two weeks. Twelve differentiation experiments were performed.

### Virus infections

Cells were infected at a multiplicity of infection (MOI) of 0.1 with cell-free VZV (vOka; Merck Sharp & Dohme Corp, Sunny Point, PA) or a lab-passaged clinical isolate of herpes simplex virus-1 (HSV) for 96 hours. Media was replaced every 2 days.

### Antibodies and immunocytochemistry

The differentiated cultures were fixed with 4% paraformaldehyde for 10 min and rinsed with PBS. Blocking was performed for one hour in blocking buffer (5% normal serum, 0.3% Triton-X 100 in PBS). Cells were incubated overnight at 4°C with the following antibodies: mouse Pax6 (Developmental Studies Hybridoma Bank [DSHB], Iowa City, Iowa), mouse nestin (Millipore), mouse βIII-tubulin (Santa Cruz Biotechnology, Santa Cruz, CA), goat Brn3a (Santa Cruz), mouse Islet-1/2 (DSHB), mouse VZV glycoprotein E (gE, Santa Cruz), mouse VZV IE62 (Santa Cruz), mouse HSV glycoprotein D (Santa Cruz). Alexa Fluor-conjugated secondary antibodies (Jackson ImmunoResearch, West Grove, PA) were used for detection of primary antibodies and 4′,6-diamidino-2-phenylindole (DAPI; Sigma-Aldrich) was used to label nuclei, and incubated for one hour at RT. When multiple mouse antibodies were used to stain the same cultures, one of the primary antibodies was directly labeled with Zenon Alexa Fluor labeling kits (Invitrogen) to avoid cross-reactivity.

### Antibodies and flow cytometry

The differentiated cultures were dissociated with Accutase, washed in PBS and blocked in 1% goat and bovine serum in PBS for one hour. The cells were fixed and permeabilized in FIX and PERM (Invitrogen) and the following primary antibodies were added for 30–45 min at RT: βIII-tubulin Alexa 647 (BD Pharmingen), goat peripherin (Santa Cruz), and rabbit Brn3a (Epitomics, Burlingame, CA). Unlabeled primary antibodies were directly conjugated with Zenon Alexa Fluor labeling kits. The cells were washed in 1% goat and bovine serum and resuspended in FACS Fix (2% paraformaldehyde in PBS). All flow cytometry was performed on a LSRII (BD Biosciences) and analyzed using FlowJo (Tree Star Inc, Ashland, OR). Autofluorescence was gated out using the violet channel.

### Microscopy

Immunocytochemistry was observed with a Nikon TS100 fluorescence microscope (Nikon Instruments, Melville, NY) and images were captured with a Nikon DS-Qi1 cooled camera head. Images were analyzed with the Nikon NIS-Elements software.

### Electrophysiology

Whole-cell current-clamp recordings of action potentials from cultured neurons were performed using methods similar to the those described by Pomp, et al [27]. Cultures were superfused with artificial cerebral spinal fluid (ACSF – concentrations in mM – 120 NaCl, 3.5 KCl, 2.5 CaCl2, 1.3 MgSO4, 1.25 NaH2PO4, 26 NaHCO3 and 10 D-glucose, saturated with 95% O2 and 5% CO2). Neurons were identified visually by infrared differential interference contrast (IR-DIC). Recording micropipettes (5–10 MΩ), pulled from thick-walled borosilicate glass (1.5 mm outer diameter, 0.85 mm inner diameter, WPI Sarasota, FL), were filled with (in mM): 122.5 Cs-gluconate, 17.5 CsCl, 10 HEPES (CsOH), 0.2 Na-EGTA, 2 Mg-ATP, 0.3 Na-GTP, and 8 NaCl at pH 7.3. At least 5 minutes elapsed between seal formation and data collection. All neurons were maintained with seal resistances of 5–10 GΩ and series resistances ≤40 MΩ. Voltage recordings were filtered at 2 kHz (8-pole Bessel) and digitized at 10 kHz into a Windows-PC computer using a MultiClamp 700A patch-clamp amplifier, a Digidata 1320A data acquisition board, and the PCLAMP 10 software suite (Molecular Devices, Sunnyvale, CA). Offline data analysis was carried out with Clampfit (part of PCLAMP).

### Reverse transcription quantitative real-time PCR (RT-qPCR)

RNA was extracted from undifferentiated and differentiated iPS cells using the RNeasy Micro Kit (Qiagen, Valencia, CA). Twenty-five ng of RNA were reverse transcribed with random primers using the High-Capacity cDNA Reverse Transcription Kit (Life Technologies) and the following cycling conditions: 25°C for 10 min, 37°C for 120 min, 85°C for 5 min. The cDNA was amplified by qPCR using Brn3a- or GAPDH-specific primers (Brn3a forward: 5′ CGT ACC ACA CGA TGA ACA GC 3′; Brn3a reverse: 5′ AGG AGA TGT GGT CCA GCA GA 3′; GAPDH forward: 5′ GAG AAC GGG AAG CTT GTC A 3′; GAPDH reverse: 5′ GAG GCA TTG CTG ATG ATC TTG 3′) on a Rotor Gene-3000 (Qiagen) with the following cycling conditions: 96°C for 2 min, 50 cycles: [95°C for 10 sec, 64°C for 15 sec, 72°C for 20 sec]. The PCR products were visualized on a 3% agarose gel run at 150 V for 40 min following by staining with ethidium bromide. Brn3a Ct values were normalized to GAPDH to calculate ΔCt. The relative Brn3a expression in undifferentiated and differentiated iPS cells was analyzed using the ΔΔCt method [33]. Ct values were measured in triplicate and all values represent three biological replicates.

### Fluorescent focus assay

To test for the presence of infectious virus, supernatants from infected differentiated iPS cell cultures were collected at 96 hours post-infection, clarified by spinning at 10,000 rpm for 2 min and transferred onto confluent MRC5 fibroblasts. Supernatants from uninfected cultures were also collected as controls. Six days later, MRC5 fibroblasts were fixed and stained with antibodies to either IE62 or gE. The assay was performed in triplicate, and the data represent the mean number of foci ± SEM.

### Statistical methods

Data were analyzed by using GraphPad Prism software (GraphPad Software, San Diego, CA). Differences in Brn3a and Islet-1 expression were statistically analyzed by unpaired *t*-test.

## Results

### Differentiation of human iPS cells to sensory neurons

**Figure 1:**
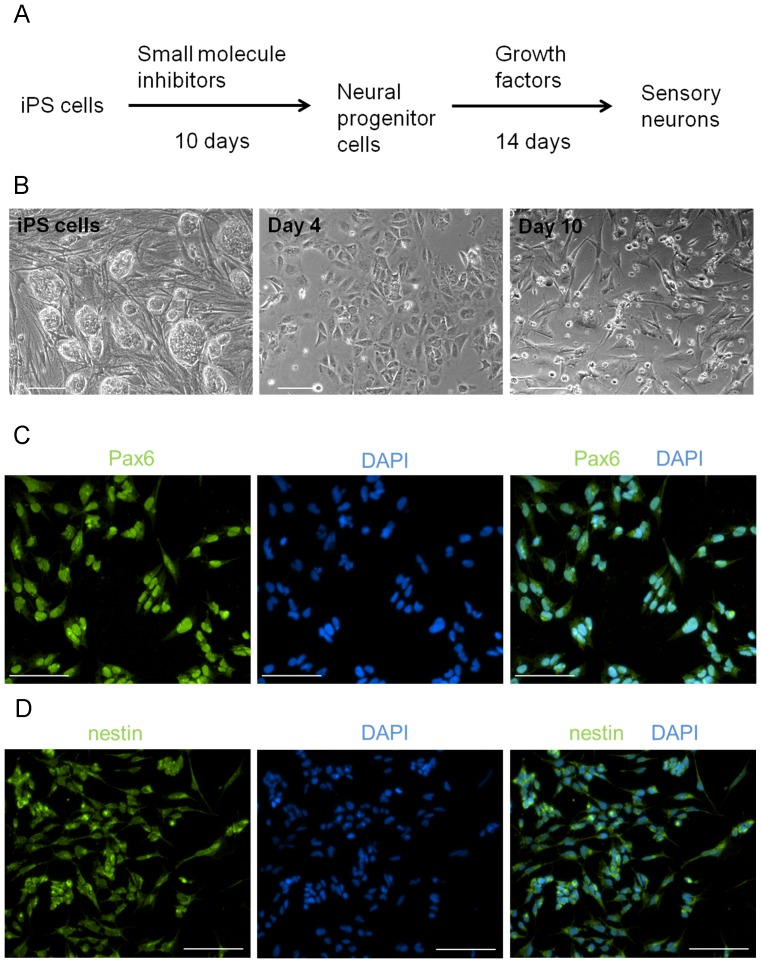
Conversion of human iPS cells to neural progenitor cells. (A) Outline of the differentiation protocol. Human iPS cells were dissociated and plated on Matrigel-coated plates. For the first 10 days, they were exposed to small molecule inhibitors, followed by culturing for two weeks in growth factors. (B) Brightfield images of iPS cells after 4 and 10 days of exposure to small molecule inhibitors. After 10 days, iPS cells expressed (C) Pax6 and (D) nestin, markers of neural progenitor cells. Nuclei were visualized with DAPI. Scale for all images is 100 um.

Our differentiation protocol, which consists of two distinct steps, takes approximately one month to complete (Figure 1A). First, iPS cells were exposed to the small molecule inhibitors (SMIs) SU5402, RO4949097, CHIR99021, A83-01 and LDN-193189, as well as retinoic acid, for 10 days. Treatment of iPS cells with SMIs resulted in a flat, epithelial-like monolayer by day 4 (Figure 1B). By day 10, a proportion of cells had died, and those remaining took on a more three-dimensional morphology (Fig. 1B) and expressed the neural progenitor markers Pax6 (Figure 1C) and nestin (Figure 1D). At this point, the cells did not express Brn3a (data not shown), a transcription factor that is detected as soon as a neuron commits to a sensory fate, and is necessary for the development and survival of neurons in sensory ganglia [34]–[36].

**Figure 2:**
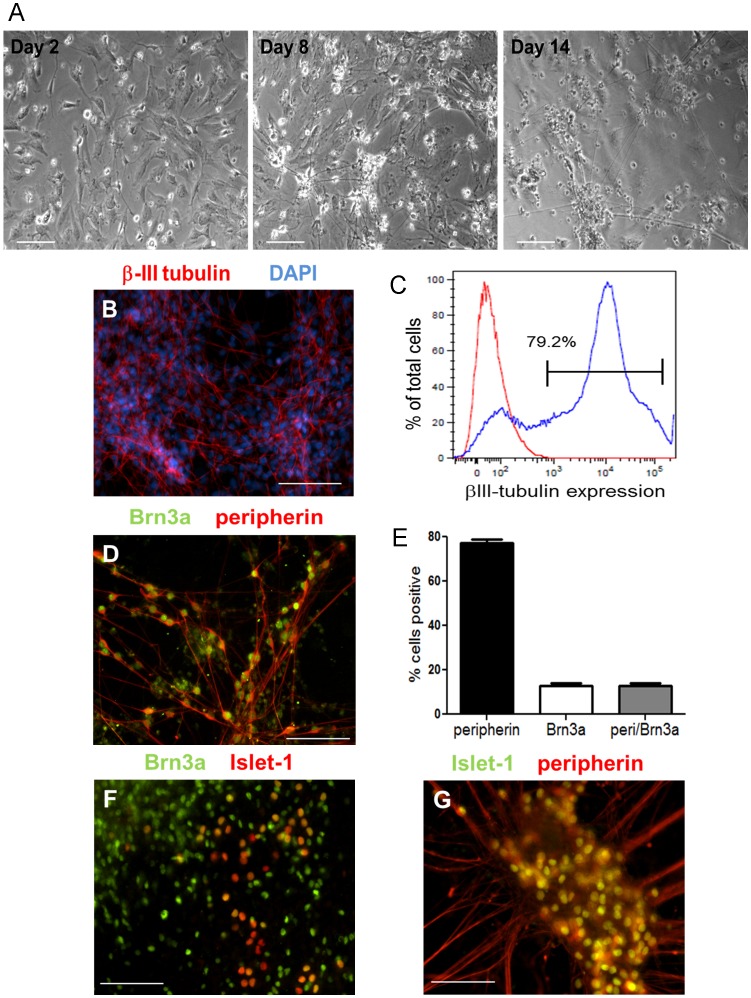
Differentiation of neural progenitor cells to sensory neurons. (A) Brightfield images of neural progenitor cells on days 2, 8 and 14 in media containing growth factors. (B) Twenty-four days after initiating differentiation of iPS cells, neurons expressed the neuronal marker βIII-tubulin. Nuclei were visualized with DAPI. (C) Approximately 80% of differentiated iPS cells are βIII-tubulin+ as determined by flow cytometry. The red tracing represents unstained cells and the blue tracing represents cells stained with an antibody to βIII-tubulin. (D) Cells also expressed peripherin and Brn3a, markers of sensory neurons. (E) The percentage of peripherin+, Brn3a+, and peripherin+/Brn3a+ cells was determined by flow cytometry. Data shown are the mean ± SEM. Co-expression of Islet-1 with (F) Brn3a and (G) peripherin. Scale for all images is 100 um.

Next, we exposed the neural progenitor cells to the growth factors NGF, BDNF, NT-3, and GDNF, as well as dibutyryl cAMP and ascorbic acid, which facilitated their conversion to sensory neurons. We observed a dramatic change in the morphology of neural progenitor cells during this period, which included the formation of colonies and the extension of multiple, long neurites (Figure 2A). After two weeks in the growth factors, we examined the cultures to determine the consequence of our differentiation protocol on the cellular composition. We observed that 80% of the differentiated cultures contained βIII-tubulin+ cells, a microtubule protein expressed by neurons (Figures 2B and 2C).

Next, we investigated whether our differentiation conditions were sufficient to generate sensory neurons. The co-expression of Brn3a with peripherin, a filament protein expressed in neurons of the peripheral nervous system, is commonly used to identify sensory neurons [18], [27], [28], [37]. By immunocytochemistry, we observed many Brn3a+/peripherin+ cells (Figure 2D), which we quantified to account for at least 15% of each culture by flow cytometry (Figure 2E). The majority of Brn3a+ cells were also positive for peripherin (Figure 2E). Furthermore, we also observed numerous cells that co-expressed Islet-1 and Brn3a (Figure 2F), which together regulate the transition from progenitor to differentiated sensory neuron [38]–[42], as well as cells that co-expressed Islet-1 and peripherin (Figure 2G).

**Figure 3:**
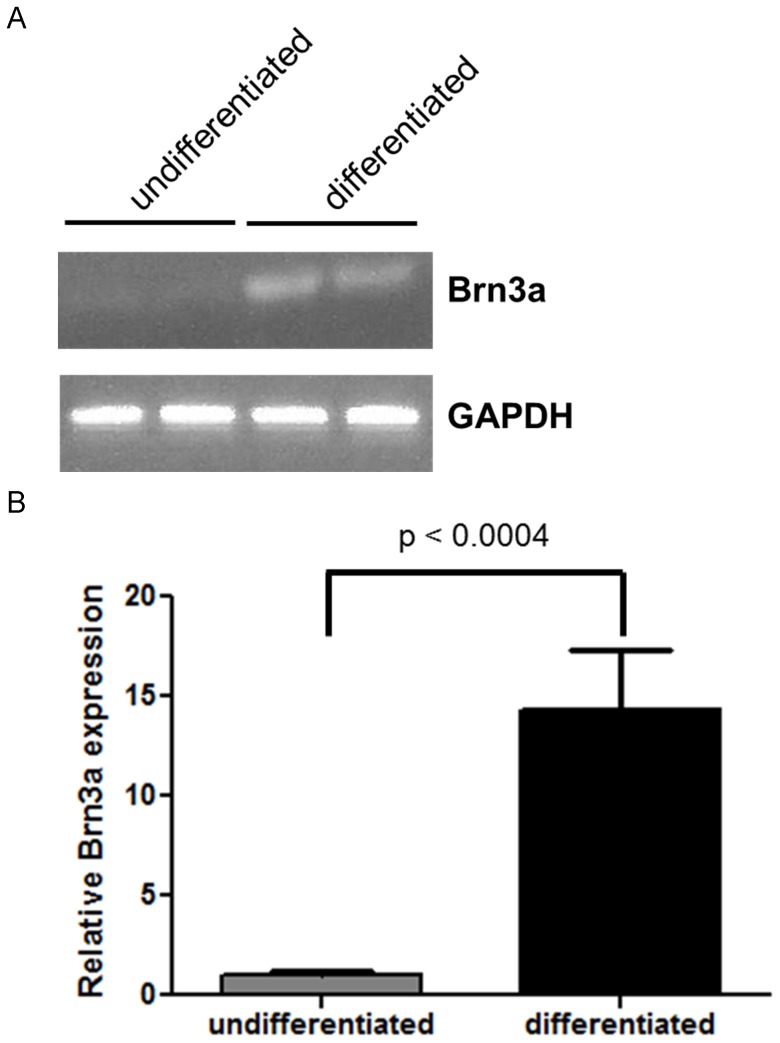
Brn3a expression is increased in differentiated iPS cells. RT-qPCR of Brn3a and GAPDH mRNA was performed. (A) Gel electrophoresis of Brn3a and GAPDH expression in undifferentiated and differentiated iPS cells. The PCR products obtained after 34 cycles was run on a 3% agarose gel. (B) The relative Brn3a gene expression in undifferentiated and differentiated iPS cells (unpaired t test, p<0.0004). Data shown are mean ± SEM of three biological replicates.

**Figure 4:**
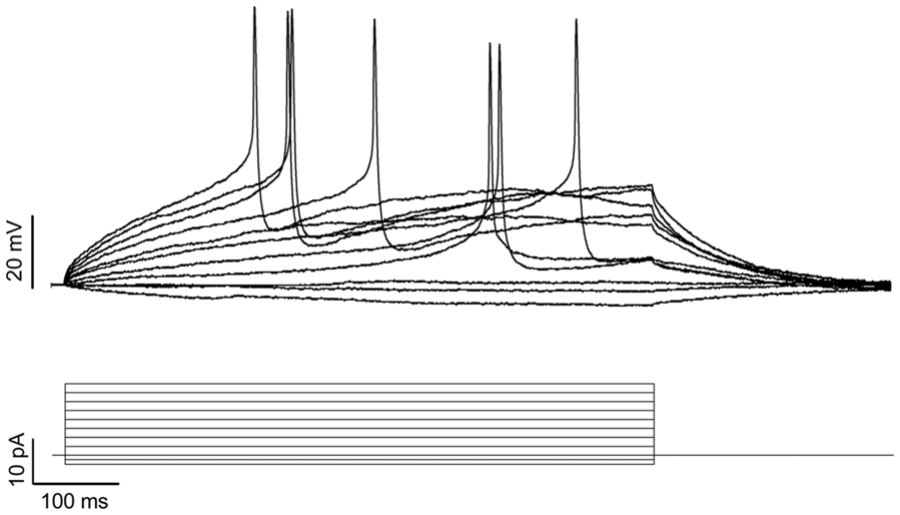
Neurons derived from iPS cells support action potentials. Whole-cell patch-clamp recordings were made in current-clamp mode from the soma of neurons. Shown above are representative traces recorded from one neuron that generated action potentials in response to depolarization. The stimulus protocol is depicted in lower traces. Membrane potentials were recorded from four neurons.

The immunocytochemistry findings were confirmed by analyzing Brn3a gene expression by RT-qPCR (Figure 3). There was >14-fold induction of Brn3a expression in differentiated as compared to undifferentiated iPS cells (p<0.0004). To ensure that the induced neurons were functional, whole-cell patch-clamp recordings were made from four neurons, each of which generated action potentials in response to depolarization (Figure 4).

### Undifferentiated iPS cells do not support VZV infection

**Figure 5:**
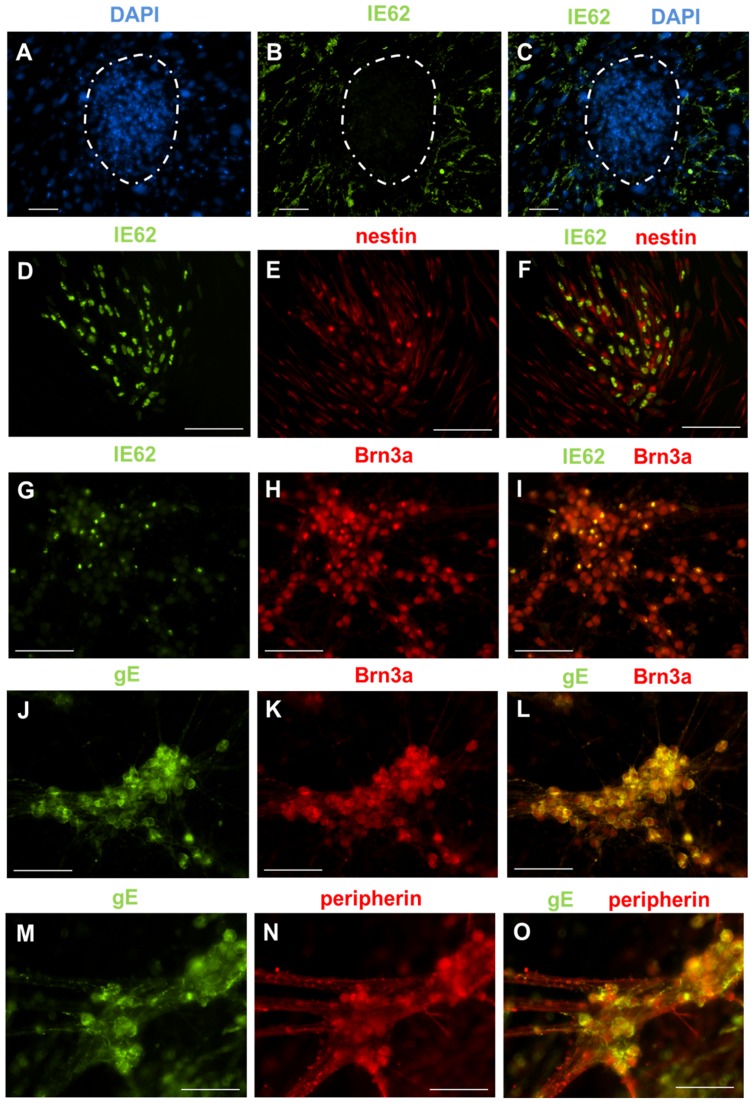
VZV infects iPS cell-derived neural progenitor cells and sensory neurons, but not undifferentiated iPS cells. (A–C) Undifferentiated iPS cell cultures were infected with cell-free VZV at a MOI of 0.1 for 96 hours. iPS cells grow as colonies (dotted white circles) on a monolayer of non-dividing fibroblasts. (A) DAPI staining identifies the iPS cell colonies. (B) Immunostaining for IE62 revealed that only the monolayer of fibroblasts supported VZV infection. No IE62 staining could be observed in the iPS cell colonies. (C) Merge of panels A and B. (D–F) Neural progenitor cells infected with cell-free VZV at a MOI of 0.1 for 96 hours. Staining for (D) IE62 and (E) nestin. (F) Co-expression of IE62 and nestin indicated that neural progenitors cells can be infected by VZV (merge of panels D and E ). (G–O) Differentiated iPS cell cultures containing sensory neurons were infected with cell-free VZV at a MOI of 0.1 for 96 hours. Immunostaining for Brn3a (H, K), peripherin (N), IE62 (G) and gE (J, M) revealed that Brn3a+ cells co-expressed IE62 (I) and gE (L). Peripherin+ cells also co-expressed gE (O) and IE62 (data not shown). Collectively, these data show that sensory neurons can be infected by VZV. Scale for all images is 100 um.

The recent observations that undifferentiated ES cells do not support VZV infection and that VZV infection was observed only after the generation of neurospheres [43] prompted us to determine the consequences of VZV infection on iPS cells at different stages of differentiation. Undifferentiated iPS cells have a distinct architecture, and grow as colonies on a monolayer of non-replicating fibroblasts (Figure 1B). This distinguishing property was helpful in determining whether iPS cells could be infected by VZV. When undifferentiated iPS cells were infected with cell-free VZV, only the supporting fibroblasts expressed the viral protein IE62, suggesting that undifferentiated iPS cells did not support VZV infection (Figures 5A–C).

### Neural progenitor cells and sensory neurons can be infected by VZV

In contrast to undifferentiated iPS cells, neural progenitor cells were readily infected by VZV, as they expressed both IE62 (Figures 5D–F) and gE (data not shown). Finally, we infected the differentiated cultures containing sensory neurons. We observed that non-neuronal cells exhibited cytopathic effects (CPE) but found that Brn3a+/peripherin+ sensory neurons remained healthy and viable despite being infected by VZV, as evidenced by the expression of IE62 and gE (Figures 5G–O). To determine whether the expression of viral proteins resulted in productive infection, supernatants were collected from iPS cell cultures containing sensory neurons that had been infected for 96 hours and transferred to a monolayer of MRC5 fibroblasts. An average of 39±9 fluorescent foci/mL was detected in the supernatants, demonstrating release of infectious virus.

### HSV infects undifferentiated iPS cells, neural precursor cells, and sensory neurons

**Figure 6:**
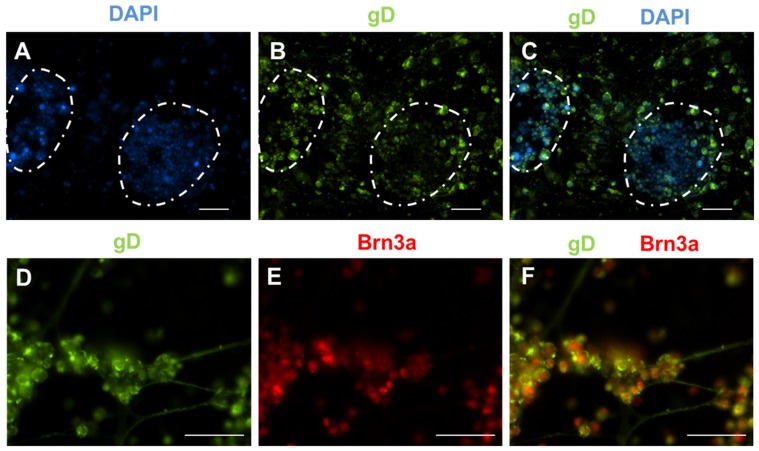
HSV infects iPS cells at all stages of differentiation. Cells were infected with cell-free HSV at a MOI of 0.1 for 96 hours. (A) DAPI staining identifies the iPS cell colonies (highlighted by the dotted white lines). (B) Immunostaining for gD revealed that both iPS cells and the underlying fibroblasts supported HSV infection, as evidenced by abundant gD expression. (C) Merge of panels A and B. (D–F) Sensory neurons derived from iPS cells also supported HSV infection. Staining for gD (D) and Brn3a (E). (F) Merge of panels D and E. Scale for all images is 100 um.

We tested whether the iPS cells at different stages of differentiation could also support infection by a related alphaherpesvirus, HSV. In contrast to what we observed for VZV, we noted robust infection of undifferentiated iPS cells by HSV, characterized by abundant expression of the viral glycoprotein gD (Figures 6A–C). Infection of neural progenitor cells was so cytotoxic that there were no viable cells left for staining (data not shown). Finally, we observed that sensory neurons in differentiated cultures could also be infected by HSV, which was confirmed by the expression of gD (Figures 6D–F). We noted abundant CPE that mainly affected non-neuronal cells, similar to what we observed with VZV infection.

## Discussion

We describe the first in vitro model of human iPS cell-derived neurons, specifically defined as sensory, that support both VZV and HSV infection. While there have been other studies directed at generating sensory neurons from either ES or iPS cells, the majority of them focused on modeling peripheral nervous system diseases, such as familial dysautonomia [25]–[28]. The more recent neuronal models developed specifically for the study of VZV did not emphasize the explicit generation or characterization of sensory neurons, which is one of the major reservoirs of latent VZV in humans [17], [18]. Furthermore, in prior studies, only MAP2α+ [17] or βIII-tubulin+ [18] cells, and not Brn3a+/peripherin+ sensory neurons, were shown to be infected by VZV.

In our initial effort to derive sensory neurons from iPS cells, we utilized the neurosphere approach. However, we observed that there was a loss of neurogenic potential with sequential passages of neurospheres, resulting in more gliogenesis, which was previously reported [27]. More recently, small molecule inhibitors (SMIs) that inhibit SMAD signaling have been used for the differentiation of ES and iPS cells to rapidly generate specific CNS cell types [44]–[47]. This approach, which bypasses the use of stromal cell lines or the generation of neurospheres, directly yields neural progenitor cells from which specific CNS cell types can be derived. Therefore, we focused on using SMIs to facilitate generation of sensory neurons.

Building upon the previous knowledge of the function of SMIs, we inhibited SMAD with LDN193189, a potent inhibitor of BMP-mediated SMAD signaling [48], and A83-01, a selective inhibitor of activin-mediated SMAD signaling, which is more potent than the commonly used SB431542 [49]. Glycogen synthase kinase-3 (GSK3) is an enzyme that functions in many signaling pathways, including those involved in development. Inhibitors of GSK3, such as CHIR99021 used in our protocol, enhance neurogenesis [50], [51]. We also included RO4929097, a member of the γ-secretase inhibitor family that accelerates neural induction by interfering with Notch signaling [46], [52]. We found that this combination of SMIs efficiently altered signaling pathways which facilitated the differentiation of iPS cells toward neural progenitor cells and subsequently sensory neurons. Our use of multiple SMIs is supported by the recent report by Chambers, et al., where they showed that by using a combination of five similar SMIs, they were able to drive differentiation of pluripotent stem cells into nociceptors, a sensory neuron subtype [53].

While our manuscript was in preparation, Dukhovny, et al. [43] reported that HSV was capable of infecting human ES cells as well as neural progenitors and neurons. They found that VZV did not infect ES cells but was capable of infecting neurons beginning at the neurosphere stage of differentiation. We found that VZV infected human iPS cell-derived sensory neurons and neural progenitor cells, but did not infect undifferentiated iPS cells. By contrast, undifferentiated iPS cells exposed to HSV were infected, similar to the findings in ES cells by Dukhovny, et al. [43]. The inability of VZV to infect iPS cells may be explained by one of the following: 1) iPS cells do not express the receptor(s) required for viral entry, or 2) iPS cells allow viral entry but do not permit VZV replication. Dukhovny, et al. reported that BAC-derived VZV transfected into ES cells did not replicate [43], although this does not rule out the possibility that VZV was unable to bind and enter the cell. They also stated that the inability of VZV to enter ES cells is not due to the lack of insulin-degrading enzyme (IDE), which has been proposed as a possible receptor for viral entry [54], as RT-PCR analysis of ES cells revealed abundant transcripts for this gene. However, they did not look for the surface expression of IDE, nor did they determine whether the cation-independent mannose-6-phosphate receptor (M6PR-ci), a known receptor for VZV entry [55], [56], was expressed on ES cells. Further studies regarding the surface expression of IDE and M6PR-ci will provide more insight into their role in VZV entry into iPS and ES cells. In contrast, neural precursor cells were readily infected by VZV. These cells are comparable to the neurospheres described by Dukhovny, et al. [43], which were also shown to support VZV infection. We also found that VZV infected our iPS cell-derived sensory neurons. The neurons could have been directly infected by the cell-free inoculum or secondarily through cell contact with infected non-neuronal cells. The observation that neurons survived VZV infection is in agreement with those reported by other groups [17], [57], [58], indicating that these cells exhibit unique properties that may explain why they support the establishment and maintenance of VZV latency. We also were able to detect infectious virus in the supernatants from the differentiated iPS cell cultures containing sensory neurons. The mean number of 40 foci/mL that was detected in our assay is similar to the number of plaques previously reported by Gowrishankar, et al. in supernatants from cultured human dorsal root ganglia [12]. In contrast to VZV, the receptors utilized by HSV for entry are abundantly expressed on many cells types [59], including ES cells [60]. Since HSV readily infects undifferentiated iPS cells, it is highly likely that these cells also express HSV receptors. Although animal models are available that support the entire HSV life cycle, they still have shortfalls when it comes to testing drug candidates [61], as it would be most ideal to test these therapeutics in a relevant host. Our model of iPS-derived sensory neurons would allow novel candidate antivirals to be tested in human cells in culture.

While our model should increase our knowledge of alphaherpesvirus pathogenesis, we appreciate that there are limitations. The differentiation protocol described yields a mixed population of cells, of which 15% have characteristics of sensory neurons. We can identify these cells by immunocytochemistry, which allow certain studies to be performed, but prevents other studies that require pure populations of cells, such as immunoblotting and PCR. Also, while we can detect infectious virus from our differentiated cultures, we cannot specify how much is being produced by sensory neurons. We are in the process of further characterizing these neurons to determine if they possess specific functional or phenotypic characteristics and whether VZV has a predilection for one type of neuron over another. Alternatively, the heterogeneous nature of the cultures would allow studies of different neuronal types as well as studies of neuronal cells in the context of other cells present in ganglia.

In summary, we show for the first time that neurons derived from human iPS cells, which bear multiple markers of sensory neurons, can support both VZV and HSV infection. Furthermore, our protocol allows for the rapid and reproducible generation of sensory neurons without the requirement for ES cells. This model will serve as a useful tool for future studies of VZV and alphaherpesvirus pathogenesis, and as a platform for screening novel therapeutics.
